# Amino Acids Supplementation Affects Sustainability of Productive and Meat Quality, Survivability and Nitrogen Pollution of Broiler Chickens during the Early Life

**DOI:** 10.3390/life12122100

**Published:** 2022-12-14

**Authors:** Youssef A. Attia, Mohammed A. Al-Harthi, Manal E. Shafi, Nisreen M. Abdulsalam, Sameer A. Nagadi, Jinquan Wang, Woo K. Kim

**Affiliations:** 1 Sustainable Agriculture Research Group, Department of Agriculture, Faculty of Environment Sciences, King Abdulaziz University, Jeddah 80269, Saudi Arabia; 2 Sustainable Agriculture Research Group, Department of Biological Sciences, Zoology, Faculty of Sciences, King Abdulaziz University, Jeddah 21589, Saudi Arabia; 3 Sustainable Agriculture Research Group, Department of Food and Nutrition, Faculty of Human Sciences and Design, King Abdulaziz University, Jeddah 21589, Saudi Arabia; 4 Sustainable Agriculture Research Group, Department of Poultry Science, University of Georgia, Athens, GA 30602-2772, USA

**Keywords:** broilers, amino acid supplementation, sustainability, growth performance, carcass yield, meat quality, nitrogen excretion

## Abstract

The response to amino acid (AAs) supplementation on broiler production, carcass and meat traits and nitrogen in the excreta during the early growth period was evaluated. Two experiments were performed during 1–28 d (245 birds, experiment 1) and 1–21 d of age (455 birds, experiment 2). In both experiments, the positive control (PC) diet had 22.5% crude protein (CP) and the negative control group (NC) diet had around 18% CP with the same methionine (Met) plus lysine (Lys) concentration as the PC diet. In experiment 1, the NC diet was fed to the other five groups supplemented with synthetic amino acids, such as L-arginine (Arg), L-threonine (Thr), L-valine (Val), L-isoleucine (Ile) or all these AAs, respectively. In experiment 2, the NC diet was formulated to contain 18% CP with either corn–soybean meal and animal protein or with only vegetable protein. Both NC diets were offered to the other ten groups with synthetic amino acids such as L-Arg, L-Thr, L-Val, L-tryptophan (Trp) or a combination of all these AAs plus L-isoleucine (Ile), respectively. In conclusion, broilers fed 18% CP supplemented with DL-Met plus L-Lys showed lower performance and a European production efficiency value (EPEV); Arg, Thr and Val addition improved growth, the feed conversion ratio and EPEV of the diets containing animal protein only, but broiler performance and EPEV was lower than with PC, indicating that DL-Met, L-Lys, L-Arg, L-Thr and L-Val supplementation may be limited in low-protein diets. Furthermore, a low-protein diet supplemented with amino acids did not affect the survivability of broilers up to 28 days of age.

## 1. Introduction

The use of a low-crude-protein diet fortified with essential amino acids is a part of a precision feeding and has gained commercial acceptance to improve protein utilization, minimize environmental impact and thus increase the sustainability of broiler production, because it reduces the cost of production [1,2,3]. Protein accounts for the second most expensive nutrient in poultry diets, following metabolizable energy [4,5]. In the typical corn–soybean meal diet, approximately 80 and 60% of dietary protein is digested and utilized by broilers and layers, respectively [6,7]. The undigested protein is excreted into the environment, causing ammonia accumulation in poultry houses. The nitrogen run off from poultry litter used as a fertilizer causes the overstimulation of the growth of aquatic plants and algae in groundwater [8,9]. In addition to the excessive N excreted leading to environmental issues, undigested dietary protein has been reported to induce necrotic-enteritis-related symptoms by hindering gut fermentation [10,11]. Thus, feeding a low-crude-protein diet is a feasible strategy to address the environmental and economic concerns related to nitrogen excretion [12,13,14,15,16]. Moreover, the recent commercial availability of the essential amino acids, including arginine, threonine, tryptophan and valine, made it possible to further reduce the crude protein levels in broiler diets [13,17,18,19,20,21]. In addition, the 2021 global supply chain crisis due to the COVID-19 pandemic created a shortage and uncertainty for the supply of available ingredients, particularly of crystalline amino acids, on the market for broiler production, and the conditions became even worse following the Russia–Ukraine conflict [21,22,23,24].

Therefore, it is crucial to test the effect of single essential amino acids and their combination on broiler performance and meat yield. However, to date there is limited research focusing on single-amino-acid supplementation in a low-protein diet, since most of the research used two or more amino acids [25,26,27].

Broilers fed a low crude protein meeting essential amino acids requirements have been reported to yield an equivalent performance and carcass protein compared to a conventional protein diet [28,29,30]. However, Dean et al. [31] reported that broiler performance deteriorates due to a limited availability of amino acids when a low-protein diet with essential amino acids is fed. These inconsistent results were identified because of a potential glycine shortage in the low-protein diet [27,32].

Thus, the main objective of the present study was to investigate the effect of satisfying the essential amino acids requirements for methionine+lysine, arginine, threonine, valine, isoleucine or tryptophan individually or in combinations on the sustainability of broiler growth performance, carcass yield, meat quality and nitrogen excretion in the early growth period. The present work consists of two experiments: the first aiming to investigate the effect of essential amino acid supplementation on a low-protein diet (18.3 vs. 22.5% CP) during 1–28 days of age. As there was a lack of a positive effect from amino acids supplementation in a low-protein diet on growth performance in experiment 1, a second experiment was conducted, in which there were the same differences in protein level (18.3 vs. 22.5%) but with the low-protein diets were formulated to contain proteins from only vegetable or vegetable plus animal sources. This tested if a higher amino acids availability from protein animal sources could restore the growth of broilers during the early growing phase (1–21 days of age).

## 2. Materials and Methods

The Deanship of King Abdulaziz University, Saudi Arabia, approved the experimental procedures adopted in this research, under the protocol no. RG-1-155-1443 H. The protocol suggested the general humane treatment of animals that does not cause distress, suffering, pain or harm, as reported by the Royal Decree number M59 in 14/9/1431H and institutional approval code ACUC-22-1-2.

### 2.1. Experimental Design

The ingredients and chemical–nutritional characteristics of the diets used in experiments 1 and 2 are shown in Table 1. In both experiments, the tested diets were formulated to meet Arbor Acers broiler requirements as reported in the management handbook [33] and by NRC [34]. Chemical–nutritional characteristics were determined according to AOAC [35] or calculated according to NRC [34].

In experiment 1, a total of 245 day-old Arbor Acres male broiler chicks were randomly allocated into seven dietary treatments with five replicates of 7 birds each. The seven dietary treatments were: (1) PC: positive control diet containing 22.5% crude protein (CP); (2) NC: negative control diet containing 18.3% CP and the same level of methionine (Met)+cystine (TSAA) and lysine (Lys) as PC; (3) NC+arginine (Arg); (4) NC+threonine (Thr); (5) NC+valine (Val); (6) NC+isoleucine (Ile); (7) NC+a combination of Arg, Thr, Val and Ile. The levels of amino acids supplementation of each diet are presented in Table 2.

The synthetic/industrial amino acids were supplemented in the experimental diets in L-from except for methionine, which was supplemented in Dl- methionine.

In experiment 2, a similar design was used as for experiment 1, with PC and NC diets containing 22.5 vs 18.3% CP. Thus, a total of 455 day-old Arbor Acres male broilers were divided into 13 groups. The groups were fed as follow: (1) PC: positive control group fed a diet containing 22.5% CP; (2) NC: negative control diet containing 18.3% of CP from both animal and vegetable sources and with the same level of Met+Lys as PC; (3) NC+Arg; (4) NC+Thr; (5) NC+Val; (6) NC+Ile; (7) NC+combination of Arg, Thr, Val and Ile. (8) NCV: negative control diet containing 18.4% of CP from only vegetable source with the same level of Met+Lys as PC; (9) NCV+Arg; (10) NCV+Thr; (11) NCV+Val; (12) NCV+Ile; (13) NCV+combination of Arg, Thr, Val and Ile. Glycine was supplemented at 1% (equivalent to 1.1662% CP) to amino acid combination diets as an amino nitrogen source. Every dietary treatment consisted of five replicates with seven birds each. The levels of AA supplementation in the diets are reported in Table 2.

The amino acids profile of the experimental diets was assayed using a Hitachi–L8900 amino acid analyzer (Minato-ku, Tokyo, Japan), at the Novus International Research Centre, Missouri, USA, according to AOAC [35].

### 2.2. Animal Husbandry

Broilers were reared in battery-brooders (40 × 45 × 60 cm), with 7 birds per cage/replicate in an environmentally controlled room. Tube feeders and automatic nipple drinkers were equipped in each battery cage. Fresh water and mash feed were supplied ad libitum throughout the experiments. Birds were checked twice a day (morning and afternoon) to monitor the general health and remove the mortality. All chicks were individually weighed, wing banded at day 0 and allocated to assure a similar initial body weight. Feed and birds were weighed at the end of each trial: 28 d of age for experiment 1 and 21 d of age for experiment 2 to calculate bodyweight gain, feed intake and feed conversion ratio. The feed conversion ratio was calculated using feed intake divided by body weight gain. Protein intake was calculated based on feed intake and the analyzed protein concentrations in the diet. The protein conversion ratio was calculated using protein intake divided by body weight gain.

At the end of the trials, a total of 30 broilers in experiment 1 and 55 in experiment 2 (5 chicks from each treatment and 1 chicken per replicate) were slaughtered after being fasted overnight to evaluate the carcass yield and traits. Carcass weight was recorded after evisceration. Abdominal plus visceral fat, liver, breast meat with wings and whole legs were separated from the carcass and weighed. Skinless boneless breast meat (*Pectorals major*) was further cut for moisture, crude protein, ether extract, and ash. In experiment 2, excreta samples were collected weekly to analyze nitrogen contents. Chemical analyses for meat and excreta were according to AOAC [35] procedures, following methods no. 934.01, 954.01, 920.39, 954.18, and 942.05, for DM, CP, EE, CF, and crude ash, respectively. The European production efficiency value was calculated according to Aviagen [33].

### 2.3. Statistical Analysis

Data from both experiments were analyzed using the GLM procedure of SAS [36] using a one-way ANOVA. In experiment 2, mixed and only-vegetable protein sources groups were analyzed separately in comparison to the positive control diet. The experimental unit was the animal. All percentages were transformed to their corresponding angles’ arcsine before running the analyses. Duncan’s post hoc test was used to separate the mean differences when *p* < 0.05.

## 3. Results

The growth performance results of experiment 1 are presented in Table 3. The diet with 18.3% of CP supplemented with Lys and Met (NC) compromised body weight gain, protein intake and protein conversion ratio compared to the PC group during 1–28 days of age (*p* < 0.05). Supplementing Arg in the NC diet increased bodyweight gain compared to the NC group; however, the Arg group is still lower than the PC group (*p* < 0.05). The supplementation of Val or amino acid combinations increased body weight gain compared to the NC group (*p* < 0.05), reaching a similar level as the PC group. Additionally, supplementation with the essential amino acid combination increased the intact protein intake compared to the NC group (*p* < 0.05); however, it was still lower than the PC group (*p* < 0.05). Initial body weight, feed intake, FCR and SR were not significantly affected by different dietary protein and amino acids supplementations.

The growth performance and N excretion results of experiment 2 are presented in Table 4. Broilers fed the low-crude-protein diet at 18.3% with Lys and Met supplementation showed a lower body weight gain for both vegetable–animal and only-vegetable protein diets compared to the PC group with 22.5% CP. Supplementation with Val or amino acids combinations in a low-protein diet with meat and fish meals improved the body weight gain compared to the NC group and it reached the same level as the PC group (*p* < 0.05). Similarly, supplementation with either single amino acids or a combination in the only-vegetable diet increased the body weight gain compared to the low-protein-diet NC group; however, it was still lower than in the PC group (*p* < 0.05). Protein intake significantly decreased in low-protein-amino-acids-supplemented diets, while PCR was improved.

Initial body weight, feed intake, FCR, EN and SR were not significantly affected by different dietary protein and amino acids supplementations.

Results of the carcass yield of experiment 1 are shown in Table 5. There was no difference for dressing, breast and wings, whole legs, liver, or abdominal fat among treatments.

The carcass yield and meat composition results of experiment 2 are shown in Table 6. Supplementation of essential amino acid combination in a low-protein diet with fish and meat meals increased the dressing percentage. Moreover, broilers fed an all-vegetable NC diet or with the supplementation of Thr, Arg, Val or amino acid combination showed a higher relative spleen weight compared to the PC group (*p* < 0.05).

The survival rate in the two experiments was not significantly affected by different amino acids supplementations in low-protein diets based on maize–soybean meal diets or maize–soybean meal diets with animal protein. This shows the safe effects of low-protein diets on the survivability of broilers up to 28 days of age.

## 4. Discussion

Our findings agree with Soares et al. [4] and Abreu et al. [5], who observed that 19% CP based on commercially available feedstuffs impaired the FCR of broilers during the growing period. However, when the negative control diets were supplemented with Met+Lys, the results constituted smallest body weight gains compared to the other groups, suggesting that Met+Lys were not the only limiting amino acids during 1–28 or 1–21 days of age in experiments 1 and 2, respectively. Thus, Val or AA mix with Gly addition to the NC diet improved growth to a level like a PC diet containing animal protein supplements. The FCR was not statistically different among treatments although PC groups showed a numerically best FCR. Abreu et al. [5] reported that broiler diets containing 19% CP formulated with unusual ingredients of starch, corn gluten bran and soybean hulls and supplemented with Thr, Arg, Trp, Val and Ile displaying a similar growth performance and production factor as the PC.

In partial agreement with the present results, Jensen and Mendonca [37] reported that the addition of 0.1% or 0.2% of L-valine to the 16% CP diet did not increase weight gains or feed efficiency while reducing abdominal fat deposition: however, the addition of 0.2% L-valine improved feed efficiency. Likewise, Cuca and Jensen [38] found that addition of arginine to 20, 21 or 22% CP diets significantly increased body weight and feed efficiency to the same level as the 22.5% CP diet. The differing effects of Val and Arg increased BWG when supplemented in diets containing animal proteins such as fish and meat meals compared to solely vegetable sources may be related to the metabolic availability of the Val and Arg. It has been reported that fish meal or meat and bone meal have a higher AA availability than soybean meal except for Val [39,40]. Thus, supplementation of Val in the NC group with an animal protein source supported the BWG equivalent to the PC diet. However, wide variation in the AA digestibility of animal protein ingredients has been documented, and AA availability is highly related to the protein quality [41]. Smith [42] reported that Val and Arg availabilities in fish meal were 67.3 and 62.6%, respectively. Along the same line, Miller and Kifer [43] found that Arg addition to poor quality fish meal improved the performance of chicks. Furthermore, Baker [12] and Boorman and Burgess [44] indicated that synthetic Arg had higher availability than natural Arg. Similarly, supplementing a low-protein diet with Lys, Met, Thr and Trp was found to improve the growth of broilers [13]. The present results revealed that Val supplementation in a low-protein diet containing fish and meat meals increased growth and EPEV to the level of the positive control diet when compared to a diet containing soybean meal as the sole protein source. It is worth noting that the growth and EPEV of broilers fed diets containing corn–soybean meal and fish meat meal were better than those fed a single protein source based on soybean meal only; however, the FCR was similar between animal protein and corn–soybean containing diets. The better performance and EPEV observed in broilers fed an animal protein source is also associated with its higher AA availability.

Supplementation with Trp increased growth in experiment 2; however, compared to Val it was found to be less effective in the animal-protein-containing diet; however in the plant-protein-containing diet, it showed an effect comparable to other amino acids for increasing growth performance and EPEV. Griminger et al. [45] reported that dietary protein did not linearly increase the requirements by the chick for Trp; however, Rogers and Pesti [46] showed that the Trp requirements increased linearly with the increases in CP % in the diet. Maynard et al. [47] reported that reducing Trp in a diet did not reduce body weight gain during 15 to 22 d of age.

Supplementation with Ile did not increase growth or improve the FCR of low-protein diets, indicating that intact Ile may be adequate and/or the responses to Ile may be governed by antagonism with Leu due to the fact that Ile and Leu are both branched-chain amino acid [12,37,48]. Combining the four supplemented amino acids with Gly has no additive effect on growth performance over that observed with Arg or Val alone. This provided evidence that the negative control diet supplemented with Met+Lys was not limited in amino nitrogen. There were no marked variations in feed intake among the experimental groups. The results of Lipstein et al. [49] and Baker [12] indicated that broilers eat in an attempt to satisfy their protein or amino acid needs.

The protein intake was constantly higher in the PC than NC group. Even though, there were no substantial variations in protein utilization among the experimental treatments in experiment 1; however, in experiment 2, the protein conversion ratio was improved by feeding the Arg- or Val-supplemented diet. The percentage of nitrogen in the excreta was lower by 15% in the low-protein-amino-acid-supplemented diets; however, the variation within and among samples was very high (±0.62), likely accounting for the lack of significance, resulting in the absence of significance. Moran et al. [50] and Jacob et al. [51] indicated that a low-protein-amino-acid-supplemented diet decreased nitrogen excretion by 27.5%. Teekel et al. [52] found that, as dietary protein was reduced, there was less uric acid and ammonia excretion, but amino acid excretion remained constant. Jirjis et al. [53] indicated that protein level did not affect amino acids in the urine significantly, but urinary nitrogen was higher with the higher protein diet. A meta-analysis by Alfonso-Avila et al. [54] reported that nitrogen intake and retention linearly decreased as the dietary protein level was reduced, particularly in days 0–21 of age. In addition, N efficiency increased by 2.3% for each 1% reduction in diet CP content regardless of bird age. A reduced crude-protein diet has also been reported to improve litter quality by reducing the litter moisture content [55]. All of these considerations can explain the increasing interest in reducing the CP level in the diets as a tool to improve the environmental sustainability of poultry production [56,57].

There were several cases of mortality in both experiments, but there were not significant differences among the groups, and, in addition, they were apparently not related to dietary treatments, as indicated by post-mortem investigations.

Carcass yield from the different experimental groups was essentially constant, and the absence of a negative effect from a low-protein-amino-acid-supplemented diet in dressing percentage, breast plus wings and thigh plus legs. It was observed that the percentages of abdominal fat and liver were not different among groups fed either the positive control or the negative control supplemented with any of the amino acids. These results agree with the conclusion of Fisher [58] and Attia et al. [16], who reported that abdominal fat could be reduced by increasing the total sulfur amino acids (TSAA) content in the broilers’ diet. Furthermore, the addition of Met to high (24%)- or low (17%)-protein diets improved the growth and FCR and reduced fat in the liver [59].

There were marked variations among the tested groups in spleen %, in which the negative control diet or diets supplemented with Thr, Arg, Val or Arg+Val+Trp+Ile had a significantly higher spleen percentage compared to the positive control diet. On the other hand, reducing CP while satisfying amino acids requirements had no negative effect on the survivability of broilers during early life.

Feeding an 18.3% CP diet supplemented with Met+Lys and Arg or Val can support normal carcass composition, as shown by the lack of significance in the crude protein, ether extract or moisture contents of the breast meat of the broilers. According to Baker [14], there was no effect of the dietary protein level when amino acids are adequate for normal tissue growth. The current findings are partially in line with those of Lipstein et al. [49], Leclercq et al. [60] and Leclercq [13], who concluded that lowering protein content while insuring adequate EAA (Arg, Ile, Val and Trp) permitted normal protein growth but increased lipid gain in both lean and fat lines of broilers, showing that lipid and protein deposition can be controlled independently and that there is no antagonism between these two meat components. Bunchasak et al. [61] observed that carcass protein and abdominal fat content were not affected by reducing intact protein from 21 to 17% when supplemented with Met and Lys to stratify these essential amino acid requirements. Van Harn et al. [55] also reported that a low-protein diet did not compromise the carcass yield. This is supported by the current results, with a lack of any significant effects of a low-protein-amino-acid-supplemented diet on dressing and carcass composition.

## 5. Conclusions

Broiler chickens fed 18.3 CP diets supplemented with Met+Lys showed lower growth performance and EPEV than the “normal” CP diets; the supplementation of Arg, Thr and Val increased growth rate and EPEV when the low-protein diets contained proteins from animal sources. However, despite these improvements, the performance and EPEV was numerically lower than that of the PC, indicating that Met, Lys, Arg, Thr and Val may be limited in the 18.3% CP diet. Thus, further research is needed to improve the sustainability of broilers fed low-protein diets supplemented with essential and non-essential amino acids during the early growth phase even through feeding low-protein diets decreased N excretion, which would potentially help with an improved environmental condition. Moreover, a low-protein diet enhanced by amino acids addition did not affect the survivability of broilers during their early life.

## Figures and Tables

**Table 1:** Composition and calculated, determined analyses of the experimental diets.

	Experiment 1		Experiment 2		
Ingredients, %	PC	NC	PC	NC	NCV
Yellow corn	53.00	66.60	57.45	66.77	62.14
Soybean meal 48% CP	36.30	22.30	33.20	24.80	31.20
Fish meal	2.00	2.00	2.00	2.00	0.00
Meat meal	2.00	2.00	2.00	2.00	0.00
Soybean oil	3.50	1.88	2.70	1.32	2.60
Dicalcium phosphate	1.00	1.10	1.00	1.10	1.80
Limestone	0.86	0.87	0.85	0.87	1.10
Premix ^1^	0.25	0.25	0.25	0.25	0.25
NaCl	0.30	0.30	0.30	0.30	0.30
DL-methionine	0.16	0.29	0.19	0.26	0.28
L-lysine	0.03	0.47	0.06	0.33	0.33
L-isoleucine	0.00	0.00	0.00	0.00	0.00
Sand	0.60	1.94	0.00	0.00	0.00
Chemical-nutritional characteristics					
ME kcal/ kg ^2^	3001	3004	3000	3005	3005
Crude protein, % ^3^	22.50	18.29	22.50	18.29	18.36
Methionine, % ^3^	0.54	0.61	0.56	0.60	0.58
TSAA, % ^3^	0.90	0.90	0.90	0.90	0.90
Lysine, % ^3^	1.31	1.31	1.25	1.25	1.25
Threonine ^3^	0.96	0.77	0.93	0.81	0.82
Arginine, % ^3^	1.34	1.03	1.33	1.09	1.14
Valine, % ^3^	1.06	0.84	1.02	0.88	0.86
Isoleucine, % ^3^	0.91	0.72	0.87	0.80	0.74
Tryptophan, % ^2^	0.33	0.23	0.31	0.25	0.27
Ca, % ^2^	0.90	0.90	1.00	0.90	0.90
Available *p*, % ^2^	0.46	0.46	0.50	0.46	0.46

^1^ Vitamin and mineral premix provides, per kilogram of diet: vitamin A, 8000 IU; vitamin E, 9 mg; menadione (as menadione sodium bisulfite), 150 IU; Vit. D_3_, 1000 ICU; riboflavin, 4.0 mg; Ca pantothenate, 10 mg; nicotinic acid, 12 mg; choline chloride, 300 mg; vitamin B_12_, 2 mg; vitamin B_6_, 1.2 mg; thiamine (as thiamine mononitrate), 2.0 mg; folic acid, 40 mg; d-biotin, 0.05 mg. Trace minerals (mg per kg of diet): Mn, 75; Zn, 40; Fe, 40; Cu, 3; Se, 0.15; iodine, 0.8 and 500 mg an antioxidant. ^2^ Calculated values according to NRC [34]; ^3^ Determined values; PC: positive control; NC: negative control; NCV: negative vegetable protein control.

**Table 2:** Amino acids supplementation for the experimental diets.

	Experiment 1		Experiment 2		
	PC	NC	PC	NC	NCV
Crude protein, %	22.50	18.3	22.5	18.3	18.4
L-Threonine, %	0.00	0.20	0.00	0.12	0.12
L-Arginine, %	0.00	0.39	0.00	0.23	0.20
L-Valine, %	0.00	0.23	0.00	0.14	0.14
L-Isoleucine, %	0.00	0.23	0.00	0.15	0.13
L-Tryptophan, %	0.00	0.00	0.00	0.05	0.03
L-Glycine, %	0.00	0.00	0.00	1.00	1.00

PC: positive control; NC: negative control; NCV: negative control with vegetable protein.

**Table 3:** Effect of amino acids supplementation on the performance of Arbor Acres broiler chicks fed a low-protein diet containing corn–soybean during 1–28 days of age (Experiment 1).

	PC	NC	NC+Thr	NC+Arg	NC+Val	NC+Ile	NC+AAs+Gly	SEM	*p* Value
IBW, g	42.3	41.7	41.9	43.6	42.6	43.4	43.8	1.18	0.77
BWG, g	1207 ^a^	1057 ^c^	1117 ^bc^	1143 ^b^	1184 ^ab^	1078 ^c^	1187 ^ab^	18.9	0.001
FI, g	2240	2242	2278	2221	2329	2342	2417	80.0	0.16
PI, g	515 ^a^	404 ^c^	410 ^c^	400 ^c^	419 ^c^	422 ^c^	435 ^b^	15.1	0.001
FCR, g/g	1.86	2.13	2.04	1.94	1.97	2.17	2.04	0.081	0.07
PCR, g/g	0.427 ^a^	0.382 ^bc^	0.367 ^bc^	0.349 ^c^	0.353 ^bc^	0.391 ^ab^	0.366 ^bc^	0.014	0.03
SR, %	97.1	97.1	97.1	97.1	97.1	97.1	100.0	2.43	0.572
EPEV	225.1 ^a^	172.1 ^c^	189.9 ^bc^	204.3 ^b^	208.4 ^ab^	172.3 ^c^	207.8 ^ab^	8.21	0.001

^a–c^ Means within a row not sharing a common a superscript differ significantly *p* < 0.05, based on Duncan’s test; PC: positive control; NC: negative control; Thr: threonine; Arg: arginine; Val: valine; Ile: isoleucine; AAs: amino acids mix; Gly: glycine; IBW: initial body weight; BWG: body weight gain; FI: feed intake; PI: protein intake; FCR: feed conversion ratio; PCR: protein conversion ratio; SR: survival rate. EPEV: European production efficiency value.

**Table 4:** Effect of amino acids supplementations on performance of Arbor Acres broiler males fed a low-protein diet containing corn–soybean with fish meal (animal protein supplement) and vegetable protein diet during 1 to 21 d of age (experiment 2).

	PC	NC	NC+Thr	NC+Arg	NC+Val	NC+Trp	NC+AAs+Gly	SEM	*p* value
NC									
IBW, g	41.0	44.4	45.4	43.9	44.1	42.3	42.8	1.31	0.50
BWG, g	813 ^a^	741 ^c^	760 ^bc^	775 ^b^	815 ^a^	773 ^bc^	790 ^ab^	8.9	0.001
FI, g	1412	1345	1365	1409	1436	1446	1376	60.3	0.85
PI, g	318 ^a^	246 ^b^	250 ^b^	258 ^b^	263 ^b^	265 ^b^	252 ^b^	11.4	0.001
FCR, g	1.74	1.82	1.80	1.81	1.77	1.86	1.76	0.07	0.29
EN, %	4.04	3.50	3.41	3.49	3.34	3.22	3.62	0.34	0.432
PCR, g/g	0.391 ^a^	0.331 ^b^	0.328 ^b^	0.333 ^b^	0.323 ^b^	0.343 ^b^	0.319 ^b^	0.013	0.001
SR, %	100.0	97.1	97.1	97.1	97.1	100.0	97.1	3.53	0.684
EPEV	222.5 ^a^	188.3 ^c^	195.2 ^bc^	198.1 ^b^	212.9 ^a^	197.9 ^bc^	207.5 ^ab^	4.87	0.001
NCV									
IBW, g	41.0	41.6	41.7	41.8	43.4	43.3	44.2	1.03	0.24
BWG, g	813 ^a^	632 ^c^	657 ^b^	673 ^b^	698 ^b^	672 ^b^	688 ^b^	7.9	0.001
FI, g	1412	1313	1323	1202	1270	1291	1283	53.4	0.22
PI, g	318 ^a^	241 ^b^	243 ^b^	221 ^b^	233 ^b^	237 ^b^	236 ^b^	9.8	0.001
FCR, g	1.74	2.08	2.01	1.80	1.90	1.92	1.87	0.09	0.17
EN, %	4.04	3.34	3.25	3.49	3.50	3.22	3.62	0.62	0.568
PCR, g/g	0.391 ^a^	0.381 ^a^	0.370 ^ab^	0.328 ^c^	0.334 ^c^	0.353 ^c^	0.343 ^bc^	0.001	0.001
SR, %	100.0	100.0	97.1	97.1	97.1	97.1	97.1	2.43	0.735
EPEV	222.5 ^a^	144.7 ^c^	151.1 ^b^	172.9 ^b^	169.9 ^b^	161.8 ^b^	170.1 ^b^	10.4	0.001

^a–c^ Means within a row not sharing a common a superscript differ significantly *p* ≤ 0.05, based on Duncan’s test. PC: positive control; NC: negative control; NCV: negative control with vegetable protein only; Thr: threonine; Arg: arginine; Val: valine; Trp: tryptophan; AAs: amino acids mix; Gly: glycine; IBW: initial body weight; BWG: body weight gain; FI: feed intake; PI: protein intake; FCR: feed conversion ratio; EN: excreta nitrogen; PCR: protein conversion ratio; SR: survival rate. EPEV: European production efficiency value.

**Table 5:** Effect of the amino acids supplementations on carcass and meat traits (% of live body weight) of Arbor Acres broiler males fed a low-protein corn–soybean diet with fish meal during 1–28 d of age (experiment 1).

	PC	NC	NC+Thr	NC+Arg	NC+Val	NC+Ile	NC+AAs+Gly	SEM	*p* Value
Dressing ^1^	61.9	61.4	62.1	61.9	61.4	61.7	61.9	1.43	0.99
Breast + wings, %	23.0	22.0	22.4	22.1	21.9	22.6	22.5	0.57	0.76
Thigh + legs, %	25.5	24.9	25.3	25.2	25.2	25.9	25.7	0.66	0.87
Liver, %	2.36	2.13	2.37	2.02	2.23	2.32	2.18	0.11	0.34
AF, %	1.31	1.57	1.53	1.47	1.56	1.37	1.33	0.14	0.64

^1^ Head, giblets, feet and eviscerate were not included; PC: positive control; NC: negative control; Thr: threonine; Arg: arginine; Val: valine; Ile: isoleucine; AAs: amino acids mix; Gly: glycine; AF: abdominal fat.

**Table 6:** Effect of amino acids supplementations on carcass yield and meat chemical composition of Arbor Acres broiler chicks fed a low-protein diet containing corn–soybean with fish meal (animal protein supplement) and vegetable protein diet during 1 to 21 d of age (experiment 2).

Parameter	PC	NC	NC+Thr	NC+Arg	NC+Val	NC+Trp	NC+AAs+Gly	SEM	*p* Value
NC									
Carcass traits									
Dressing ^1^, %	62.4 ^b^	61.3 ^b^	62.6 ^b^	63.3 ^ab^	59.5 ^b^	63.2 ^ab^	63.8 ^a^	1.06	0.02
Breast + wings, %	21.5	22.5	22.8	22.6	22.2	22.9	23.4	0.56	041
Thigh + legs, %	25.0	24.3	24.6	23.7	24.0	25.0	24.8	0.77	0.19
AF, %	1.38	0.77	0.75	1.65	1.39	1.63	1.41	0.20	0.13
Liver, %	2.75	2.73	2.68	2.33	2.64	2.49	2.57	0.13	0.36
Spleen, %	0.10	0.12	0.13	0.15	0.12	0.12	0.14	0.013	0.32
Meat chemical composition ^2^									
Dry matter, %	24.4	24.9	24.6	25.1	24.8	24.7	24.2	0.81	0.43
Crude protein, %	84.8	85.3	84.0	81.0	85.4	84.4	82.9	1.56	0.78
Ether extract, %	15.1	13.7	16.2	18.8	14.3	15.1	16.8	0.74	0.34
Ash, %	3.51	3.71	3.32	3.42	3.56	3.48	3.68	0.24	0.53
NCV									
Carcass traits									
Dressing ^1^, %	61.4	61.5	61.7	61.1	61.6	59.4	62.4	1.25	0.35
Breast + wings, %	21.5	22.1	22.5	22.0	21.2	22.4	21.2	0.84	0.92
Thigh + legs, %	25.0	25.0	25.8	24.4	25.2	25.3	25.2	1.14	0.78
AF, %	1.38	1.23	1.33	1.06	0.96	1.27	0.96	0.17	0.52
Liver, %	2.75	2.78	2.75	2.84	2.55	2.61	2.59	0.13	0.61
Spleen, %	0.10 ^c^	0.16 ^a^	0.17 ^a^	0.17 ^a^	0.14 ^ab^	0.12 ^bc^	0.14 ^ab^	0.011	0.01
Meat chemical composition ^2^									
Moisture, %	24.4	25.1	24.7	24.8	24.4	24.6	24.2	0.87	0.56
Crude protein, %	84.8	83.2	82.6	83.9	85.1	83.6	82.7	1.56	0.73
Ether extract, %	15.1	15.7	15.9	15.7	14.6	16.2	17.1	0.74	0.29
Ash, %	3.48	3.74	3.38	3.62	3.49	3.52	3.28	0.34	0.49

^1^ Head, giblets, feet and eviscerate were not included. ^2^ On a relative dry matter basis. ^abc^ Means within a row not sharing a common a superscript differ significantly *p* ≤ 0.05, based on Duncan’s test, ND, not done. PC: positive control; NC: negative control; NCV: negative control with vegetable protein only; Thr: threonine; Arg: arginine; Val: valine; Trp: tryptophan; AAs: amino acids mix; Gly: glycine; AF: abdominal fat.

## Data Availability

The data are available upon official request from the principal investigator upon the permission of the funding agent.
